# Supplementary material for: Discovery and application of insertion-deletion (INDEL) polymorphisms for QTL mapping of early life-history traits in Atlantic salmon
Source: BMC Genomics. 2010 Mar 8;11:156. doi: 10.1186/1471-2164-11-156 (PMC2838853; doi:10.1186/1471-2164-11-156)
Supplement: Additional file 2 — Information on developed 76 locus single-run INDEL panel in Atlantic salmon. Information on fluorescence labeling, primer concentrations, PCR pooling and links to alignments, INDEL motifs and GENESCAN (Burge and Karlin 1997) predictions of genes/exons are available in html format. [file 1471-2164-11-156-S2.ZIP › Additionalfile2/table9552.html]

Contig Alignment

```
Base	A B C D E F   
1	. . G . . .    
2	. . T . . .    
3	. . A A . .    
4	. . T T . .    
5	. . C C . .    
6	. . T T . .    
7	. . T T . .    
8	. . C C . .    
9	. . A A . .    
10	A A A A . .    
11	A A A A . .    
12	T T T T . .    
13	G G G G . .    
14	T T T T . .    
15	A A A A . .    
16	T T T T . .    
17	T T T T . .    
18	T T T T . .    
19	A A A A . .    
20	T T T T . .    
21	T T T T . .    
22	C C C C . .    
23	T T T T . .    
24	T T T T . .    
25	G G G G . .    
26	T T T T . .    
27	G G G G . .    
28	G G G G . .    
29	T T T T . .    
30	A A A A . .    
31	A A A A . .    
32	C C C C . .    
33	C C C C . .    
34	A A A A . .    
35	T T T T . .    
36	T T T T . .    
37	C C C C . .    
38	A A A A . .    
39	T T T T . .    
40	A A A A . .    
41	T T T T . .    
42	C C C C . .    
43	C C C C . .    
44	C C C C . .    
45	A A A A . .    
46	A A A A . .    
47	A A A A . .    
48	A A A A . .    
49	C C C C . .    
50	A A A A . .    
51	C C C C . .    
52	A A A A . .    
53	C C C C . .    
54	A A A A . .    
55	C C C C . .    
56	T T T T . .    
57	T T T T . .    
58	C C C C . .    
59	A A A A . .    
60	A A A A . .    
61	T T T T . .    
62	G G G G . .    
63	T T T T . .    
64	A A A A . .    
65	C C C C . .    
66	A A A A . .    
67	T T T T . .    
68	G G G G . .    
69	G G G G . .    
70	T T T T . .    
71	G G G G . .    
72	A A A A . .    
73	C C C C . .    
74	G G G G . .    
75	T T T T . .    
76	G G G G . .    
77	C C C C . .    
78	A A A A . .    
79	A A A A . .    
80	A A A A . .    
81	A A A A . .    
82	A A A A . .    
83	G G G G . .    
84	G G G G . .    
85	C C C C . .    
86	A A A A . .    
87	T T T T . .    
88	T T T T . .    
89	T T T T . .    
90	T T T T . .    
91	G G G G . .    
92	A A A A . .    
93	G G G G . .    
94	G G G G . .    
95	T T T T . .    
96	G G G G . .    
97	G G G G . .    
98	G G G G . .    
99	A A A A . .    
100	A A A A . .    
101	A A A A . .    
102	G G G G . .    
103	A A A A . .    
104	G G G G . .    
105	T T T T . .    
106	G G G G . .    
107	A A A A . .    
108	G G G G . .    
109	A A A A . .    
110	A A A A . .    
111	C C C C . .    
112	T T T T . .    
113	G G G G . .    
114	T T T T . .    
115	G G G G . .    
116	A A A A . .    
117	A A A A . .    
118	A A A A . .    
119	T T T T . .    
120	G G G G . .    
121	A A A A . .    
122	A A A A . .    
123	T T T T . .    
124	C C C C . .    
125	T T T T . .    
126	C C C C . .    
127	T T T T . .    
128	T T T T . .    
129	A A A A . .    
130	A A A A . .    
131	C C C C . .    
132	A A A A . .    
133	G G G G . .    
134	A A A A . .    
135	T T T T . .    
136	A A A A . .    
137	C C C C . .    
138	A A A A . .    
139	C C C C . .    
140	T T T T . .    
141	G G G G . .    
142	A A A A . .    
143	T T T T . .    
144	C C C C . .    
145	A A A A . .    
146	C C C C . .    
147	G G G G . .    
148	A A A A . .    
149	T T T T . .    
150	C C C C . .    
151	C C C C . .    
152	A A A A . .    
153	A A A A . .    
154	G G G G . .    
155	C C C C . .    
156	A A A A . .    
157	G G G G . .    
158	T T T T . .    
159	T T T T . .    
160	G G G G . .    
161	C C C C . .    
162	T T T T . .    
163	G G G G . .    
164	A A A A . .    
165	A A A A . .    
166	A A A A . .    
167	A A A A . .    
168	A A A A . .    
169	A A A A . .    
170	C C C C . .    
171	C C C C . .    
172	C C C C . .    
173	C C C C . .    
174	A A A A . .    
175	C C C C . .    
176	A A A A . .    
177	A A A A . .    
178	A A A A . .    
179	T T T T . .    
180	A A A A . .    
181	C C C C . .    
182	C C C C . .    
183	A A A A . .    
184	G G G G . .    
185	A A A A . .    
186	G G G G . .    
187	T T T T . .    
188	G G G G . .    
189	T T T T . .    
190	T T T T . .    
191	A A A A . .    
192	G G G G . .    
193	G G G G . .    
194	A A A A . .    
195	T T T T . .    
196	G G G G . .    
197	T T T T . .    
198	G G G G . .    
199	T T T T . .    
200	A A A A . .    
201	T T T T . .    
202	A A A A . .    
203	C C C C . .    
204	T T T T . .    
205	C C C C . .    
206	T T T T . .    
207	C C C C . .    
208	C C C C . .    
209	A A A A . .    
210	C C C C . .    
211	A A A A . .    
212	C C C C . .    
213	A A A A . .    
214	C C C C . .    
215	A A A A . .    
216	A A A A . .    
217	T T T T . .    
218	A A A A . .    
219	G G G G . .    
220	C C C C . .    
221	T T T T . .    
222	C C C C . .    
223	T T T T . .    
224	T T T T . .    
225	T T T T . .    
226	C C C C . .    
227	A A A A . .    
228	T T T T . .    
229	A A A A . .    
230	G G G G . .    
231	A A A A . .    
232	A A A A . .    
233	A A A A . .    
234	G G G G . .    
235	A A A A . .    
236	A A A A . .    
237	T T T T . .    
238	G G G G . .    
239	A A A A . .    
240	C C C C . .    
241	A A A A . .    
242	T T T T . .    
243	C C C C . .    
244	A A A A . .    
245	T T T T . .    
246	T T T T . .    
247	A A A A . .    
248	C C C C . .    
249	T T T T . .    
250	G G G G . .    
251	A A A A . .    
252	G G G G . .    
253	G G G G . .    
254	A A A A . .    
255	A A A A . .    
256	C C C C . .    
257	T T T T . .    
258	- - C C . .  2	 indel  
259	- - T T . .  2	 indel  
260	G G G G . .    
261	C C C C . .    
262	A A A A . .    
263	A A A A . .    
264	C C C C . .    
265	A A A A . .    
266	C C C C . .    
267	C C C C . .    
268	A A A A . .    
269	T T T T . .    
270	C C C C . .    
271	A A A A . .    
272	C C C C . .    
273	A A A A . .    
274	T T T T . .    
275	A A A A . .    
276	C C C C . .    
277	A A A A . .    
278	A A A A . .    
279	C C C C . .    
280	A A A A . .    
281	G G G G . .    
282	C C C C . .    
283	C C C C . .    
284	A A A A . .    
285	A A A A . .    
286	A A A A . .    
287	T T T T . .    
288	G G G G . .    
289	A A A A . .    
290	G G G G . .    
291	G G G G . .    
292	A A A A . .    
293	T T T T . .    
294	G G G G . .    
295	C C C C . .    
296	T T T T . .    
297	A A A A . .    
298	A A A A . .    
299	A A A A . .    
300	T T T T . .    
301	C C C C . .    
302	T T T T . .    
303	A A A A . .    
304	A A A A . .    
305	A A A A . .    
306	T T T T . .    
307	A A A A . .    
308	T T T T . .    
309	A A A A . .    
310	G G G G . .    
311	T T T T . .    
312	T T T T . .    
313	C C C C . .    
314	C C C C . .    
315	A A A A . .    
316	A A A A . .    
317	A A A A . .    
318	T T T T . .    
319	A A A A . .    
320	T T T T . .    
321	A A A A . .    
322	C C C C . .    
323	A A A A . .    
324	A A A A . .    
325	G G G G . .    
326	A A A A . .    
327	C C C C . .    
328	T T T T . .    
329	A A A A . .    
330	A A A A . .    
331	C C C C . .    
332	C C C C . .    
333	C C C C . .    
334	A A A A . .    
335	A A A A . .    
336	G G G G . .    
337	C C C C . .    
338	C C C C . .    
339	A A A A . .    
340	T T T T . .    
341	A A A A . .    
342	A A A A . .    
343	C C C C . .    
344	A A A A . .    
345	G G G G . .    
346	A A A A . .    
347	A A A A . .    
348	A A A A . .    
349	T T T T . .    
350	A A A A . .    
351	T T T T . .    
352	A A A A . .    
353	T T T T . .    
354	C C C C . .    
355	C C C C . .    
356	A A A A . .    
357	A A A A . .    
358	T T T T . .    
359	A A A A . .    
360	A A A A . .    
361	A A A A . .    
362	G G G G . .    
363	A A A A . .    
364	A A A A . .    
365	T T G G . .  2	 transversion  
366	. . A A . .    
367	. . A A . .    
368	. . T T . .    
369	. . T T . .    
370	. . C C . .    
371	. . A A . .    
372	. . T T . .    
373	. . T T . .    
374	. . T T . .    
375	. . C C . .    
376	. . A A . .    
377	. . T T . .    
378	. . A A . .    
379	. . A A . .    
380	. . A A . .    
381	. . C C . .    
382	. . A A . .    
383	. . T T . .    
384	. . T T . .    
385	. . G G . .    
386	. . T T . .    
387	. . A A . .    
388	. . A A . .    
389	. . A A . .    
390	. . G G . .    
391	. . G G . .    
392	. . T T . .    
393	. . A A . .    
394	. . A A . .    
395	. . C C . .    
396	. . C C . .    
397	. . C C . .    
398	. . A A . .    
399	. . T T . .    
400	. . A A . .    
401	. . A A . .    
402	. . A A . .    
403	. . A A . .    
404	. . T T . .    
405	. . G G . .    
406	. . T T . .    
407	. . C C . .    
408	. . T T . .    
409	. . G G . .    
410	. . C C . .    
411	. . T T . .    
412	. . A A . .    
413	. . A A . .    
414	. . A A . .    
415	. . A A . .    
416	. . G G . .    
417	. . T T . .    
418	. . T T . .    
419	. . C C . .    
420	. . C C . .    
421	. . A A . .    
422	. . A A . .    
423	. . C C . .    
424	. . A A . .    
425	. . T T . .    
426	. . A A . .    
427	. . A A . .    
428	. . T T . .    
429	. . A A . .    
430	. . C C . .    
431	. . T T . .    
432	. . G G . .    
433	. . T T . .    
434	. . A A . .    
435	. . A A . .    
436	. . T T . .    
437	. . T T . .    
438	. . G G . .    
439	. . T T . .    
440	. . G G . .    
441	. . C C . .    
442	. . T T . .    
443	. . T T . .    
444	. . T T . .    
445	. . C C . .    
446	. . A A . .    
447	. . G G . .    
448	. . T T . .    
449	. . G G . .    
450	. . C C . .    
451	. . A A . .    
452	. . A A . .    
453	. . A A . .    
454	. . T T . .    
455	. . T T . .    
456	. . A A . .    
457	. . G G . .    
458	. . T T . .    
459	. . C C . .    
460	. . T T . .    
461	. . G G . .    
462	. . T T . .    
463	. . C C . .    
464	. . T T . .    
465	. . C C . .    
466	. . C C . .    
467	. . A A . .    
468	. . T T . .    
469	. . C C . .    
470	. . C C . .    
471	. . A A . .    
472	. . A A . .    
473	. . A A . .    
474	. . A A . .    
475	. . T T . .    
476	. . C C . .    
477	. . T T . .    
478	. . C C . .    
479	. . C C . .    
480	. . A A . .    
481	. . G G . .    
482	. . T T . .    
483	. . A A . .    
484	. . C C . .    
485	. . T T . .    
486	. . G G . .    
487	. . T T . .    
488	. . G G . .    
489	. . T T . .    
490	. . G G . .    
491	. . T T . .    
492	. . G G . .    
493	. . T T . .    
494	. . G G . .    
495	. . A A . .    
496	. . G G . .    
497	. . C C . .    
498	. . A A . .    
499	. . A A . .    
500	. . C C . .    
501	. . A A . .    
502	. . T T . .    
503	. . C C . .    
504	. . T T . .    
505	. . A A . .    
506	. . C C . .    
507	. . A A . .    
508	. . C C . .    
509	. . T T . .    
510	. . T T . .    
511	. . T T . .    
512	. . A A . .    
513	. . G G . .    
514	. . A A . .    
515	. . A A . .    
516	. . A A . .    
517	. . G G . .    
518	. . T T . .    
519	. . G G . .    
520	. . C C . .    
521	. . T T . .    
522	. . T T . .    
523	. . C C . .    
524	. . C C . .    
525	. . T T . .    
526	. . T T . .    
527	. . C C . .    
528	. . A A . .    
529	. . C C . .    
530	. . C C . .    
531	. . T T . .    
532	. . T T . .    
533	. . T T . .    
534	. . A A . .    
535	. . A A . .    
536	. . C C . .    
537	. . C C . .    
538	. . T T . .    
539	. . C C . .    
540	. . A A . .    
541	. . C C . .    
542	. . C C . .    
543	. . A A . .    
544	. . C C . .    
545	. . A A . .    
546	. . G G . .    
547	. . C C . .    
548	. . T T . .    
549	. . A A . .    
550	. . C C . .    
551	. . C C . .    
552	. . T T . .    
553	. . G G . .    
554	. . T T . .    
555	. . C C . .    
556	. . T T . .    
557	. . G G . .    
558	. . T T . .    
559	. . A A . .    
560	. . T T . .    
561	. . G G . .    
562	. . T T . .    
563	. . C C . .    
564	. . T T . .    
565	. . T T . .    
566	. . A A . .    
567	. . A A . .    
568	. . A A . .    
569	. . C C . .    
570	. . A A . .    
571	. . T T . .    
572	. . T T . .    
573	. . G G . .    
574	. . A A . .    
575	. . G G . .    
576	. . C C . .    
577	. . C C . .    
578	. . A A . .    
579	. . G G . .    
580	. . C C . .    
581	. . T T . .    
582	. . C C . .    
583	. . A A . .    
584	. . G G . .    
585	. . A A . .    
586	. . T T . .    
587	. . A A . .    
588	. . T T . .    
589	. . A A . .    
590	. . T T . .    
591	. . T T . .    
592	. . A A . .    
593	. . A A . .    
594	. . A A . .    
595	. . T T . .    
596	. . C C . .    
597	. . T T . .    
598	. . C C . .    
599	. . T T . .    
600	. . A A . .    
601	. . C C . .    
602	. . C C . .    
603	. . T T . .    
604	. . A A . .    
605	. . T T . .    
606	. . T T . .    
607	. . A A . .    
608	. . A A . .    
609	. . G G . .    
610	. . A A . .    
611	. . T T . .    
612	. . T T . .    
613	. . T T . .    
614	. . G G . .    
615	. . T T . .    
616	. . G G . .    
617	. . C C . .    
618	. . C C . .    
619	. . A A . .    
620	. . G G . .    
621	. . T T . .    
622	. . T T . .    
623	. . C C . .    
624	. . C C . .    
625	. . A A . .    
626	. . C C . .    
627	. . T T . .    
628	. . G G . .    
629	. . G G . .    
630	. . C C . .    
631	. . A A . .    
632	. . G G . .    
633	. . C C . .    
634	. . T T . .    
635	. . C C . .    
636	. . A A . .    
637	. . G G . .    
638	. . A A . .    
639	. . G G . .    
640	. . A A . .    
641	. . A A . .    
642	. . T T . .    
643	. . A A . .    
644	. . A A . .    
645	. . T T . .    
646	. . C C . .    
647	. . T T . .    
648	. . G G . .    
649	. . T T . .    
650	. . T T . .    
651	. . A A . .    
652	. . A A . .    
653	. . A A . .    
654	. . G G . .    
655	. . C C . .    
656	. . T T . .    
657	. . T T . .    
658	. . A A . .    
659	. . G G . .    
660	. . C C . .    
661	. . A A . .    
662	. . T T . .    
663	. . G G . .    
664	. . C C . .    
665	. . A A . .    
666	. . C C . .    
667	. . T T . .    
668	. . G G . .    
669	. . T T . .    
670	. . A A . .    
671	. . T T . .    
672	. . A A . .    
673	. . C C . .    
674	. . G G . .    
675	. . T T . .    
676	. . T T . .    
677	. . A A . .    
678	. . A A . .    
679	. . G G . .    
680	. . G G . .    
681	. . C C . .    
682	. . C C . .    
683	. . T T . .    
684	. . T T . .    
685	. . T T . .    
686	. . C C . .    
687	. . A A . .    
688	. . A A . .    
689	. . A A . .    
690	. . G G . .    
691	. . C C . .    
692	. . A A . .    
693	. . G G . .    
694	. . C C . .    
695	. . G G . .    
696	. . T T . .    
697	. . G G . .    
698	. . A A . .    
699	. . T T . .    
700	. . T T . .    
701	. . C C . .    
702	. . T T . .    
703	. . C C . .    
704	. . C C . .    
705	. . A A . .    
706	. . A A . .    
707	. . A A . .    
708	. . T T . .    
709	. . T T . .    
710	. . A A . .    
711	. . A A . .    
712	. . C C . .    
713	. . A A . .    
714	. . T T . .    
715	. . C C . .    
716	. . T T . .    
717	. . A A . .    
718	. . A A . .    
719	. . C C . .    
720	. . C C . .    
721	. . A A . .    
722	. . T T . .    
723	. . A A . .    
724	. . T T . .    
725	. . T T . .    
726	. . G G . .    
727	. . C C . .    
728	. . A A . .    
729	. . A A . .    
730	. . C C . .    
731	. . A A . .    
732	. . A A . .    
733	. . T T . .    
734	. . A A . .    
735	. . T T . .    
736	. . A A . .    
737	. . A A . .    
738	. . C C . .    
739	. . T T . .    
740	. . T T . .    
741	. . T T . .    
742	. . T T . .    
743	. . A A . .    
744	. . T T . .    
745	. . . A . .    
746	. . . A . .    
747	. . . T . .    
748	. . . T . .    
749	. . . A . .    
750	. . . G . .    
751	. . . A . .    
752	. . . A . .    
753	. . . G . .    
754	. . . C . .    
755	. . . A . .    
756	. . . G . .    
757	. . . A . .    
758	. . . C . .    
759	. . . T . .    
760	. . . G . .    
761	. . . A . .    
762	. . . A . .    
763	. . . C C .    
764	. . . C C .    
765	. . . A A .    
766	. . . A A .    
767	. . . A A .    
768	. . . T T .    
769	. . . G G .    
770	. . . A A .    
771	. . . G G .    
772	. . . A A .    
773	. . . A A .    
774	. . . G G .    
775	. . . T T .    
776	. . . G G .    
777	. . . A A .    
778	. . . A A .    
779	. . . A A .    
780	. . . A A .    
781	. . . G G .    
782	. . . T T .    
783	. . . C C .    
784	. . . A A .    
785	. . . G G .    
786	. . . C C .    
787	. . . A A .    
788	. . . T T .    
789	. . . T T .    
790	. . . A A .    
791	. . . T T .    
792	. . . C C .    
793	. . . A A .    
794	. . . C C .    
795	. . . T T .    
796	. . . A A .    
797	. . . A A .    
798	. . . A A .    
799	. . . G G .    
800	. . . C C .    
801	. . . C C .    
802	. . . A A .    
803	. . . A A .    
804	. . . A A .    
805	. . . T T .    
806	. . . A A .    
807	. . . A A .    
808	. . . T T .    
809	. . . C C .    
810	. . . A A .    
811	. . . A A .    
812	. . . C C .    
813	. . . T T .    
814	. . . A A .    
815	. . . A A .    
816	. . . C C .    
817	. . . C C .    
818	. . . . T .    
819	. . . . T .    
820	. . . . A .    
821	. . . . C .    
822	. . . . A .    
823	. . . . A .    
824	. . . . C .    
825	. . . . T .    
826	. . . . A .    
827	. . . . A .    
828	. . . . G .    
829	. . . . C .    
830	. . . . C .    
831	. . . . G .    
832	. . . . G .    
833	. . . . C .    
834	. . . . C .    
835	. . . . T .    
836	. . . . T .    
837	. . . . C .    
838	. . . . T .    
839	. . . . T .    
840	. . . . T .    
841	. . . . C .    
842	. . . . T .    
843	. . . . C .    
844	. . . . T .    
845	. . . . T .    
846	. . . . C .    
847	. . . . A .    
848	. . . . C .    
849	. . . . A .    
850	. . . . T .    
851	. . . . T .    
852	. . . . G .    
853	. . . . T .    
854	. . . . A .    
855	. . . . T .    
856	. . . . T .    
857	. . . . C .    
858	. . . . T .    
859	. . . . A .    
860	. . . . A .    
861	. . . . G .    
862	. . . . A .    
863	. . . . A .    
864	. . . . G .    
865	. . . . G .    
866	. . . . C .    
867	. . . . T .    
868	. . . . G .    
869	. . . . T .    
870	. . . . A .    
871	. . . . C .    
872	. . . . A .    
873	. . . . G .    
874	. . . . T .    
875	. . . . C .    
876	. . . . T .    
877	. . . . G .    
878	. . . . T .    
879	. . . . T .    
880	. . . . A .    
881	. . . . G .    
882	. . . . A .    
883	. . . . A .    
884	. . . . T .    
885	. . . . G .    
886	. . . . T .    
887	. . . . A .    
888	. . . . T .    
889	. . . . G .    
890	. . . . T .    
891	. . . . C .    
892	. . . . T .    
893	. . . . G .    
894	. . . . C .    
895	. . . . A .    
896	. . . . T .    
897	. . . . G .    
898	. . . . T .    
899	. . . . A .    
900	. . . . T .    
901	. . . . T .    
902	. . . . A .    
903	. . . . T .    
904	. . . . G .    
905	. . . . T .    
906	. . . . G .    
907	. . . . A .    
908	. . . . G .    
909	. . . . G .    
910	. . . . C .    
911	. . . . A .    
912	. . . . G .    
913	. . . . T .    
914	. . . . T .    
915	. . . . C .    
916	. . . . T .    
917	. . . . T .    
918	. . . . A .    
919	. . . . T .    
920	. . . . T .    
921	. . . . G .    
922	. . . . A .    
923	. . . . T .    
924	. . . . A .    
925	. . . . T .    
926	. . . . C .    
927	. . . . T .    
928	. . . . T .    
929	. . . . C .    
930	. . . . A .    
931	. . . . C .    
932	. . . . A .    
933	. . . . A .    
934	. . . . C .    
935	. . . . G .    
936	. . . . A .    
937	. . . . G .    
938	. . . . T .    
939	. . . . G .    
940	. . . . C .    
941	. . . . T .    
942	. . . . T .    
943	. . . . T .    
944	. . . . T .    
945	. . . . G .    
946	. . . . T .    
947	. . . . A .    
948	. . . . C .    
949	. . . . C .    
950	. . . . T .    
951	. . . . G .    
952	. . . . T .    
953	. . . . G .    
954	. . . . G .    
955	. . . . A .    
956	. . . . G .    
957	. . . . C .    
958	. . . . A .    
959	. . . . C .    
960	. . . . T .    
961	. . . . A .    
962	. . . . A .    
963	. . . . A .    
964	. . . . C .    
965	. . . . A .    
966	. . . . A .    
967	. . . . T .    
968	. . . . T .    
969	. . . . G .    
970	. . . . A .    
971	. . . . G .    
972	. . . . T .    
973	. . . . A .    
974	. . . . A .    
975	. . . . G .    
976	. . . . C .    
977	. . . . A .    
978	. . . . T .    
979	. . . . C .    
980	. . . . A .    
981	. . . . G .    
982	. . . . C .    
983	. . . . A .    
984	. . . . T .    
985	. . . . C .    
986	. . . . A .    
987	. . . . G .    
988	. . . . T .    
989	. . . . A .    
990	. . . . G .    
991	. . . . A .    
992	. . . . C .    
993	. . . . A .    
994	. . . . C .    
995	. . . . A .    
996	. . . . T .    
997	. . . . A .    
998	. . . . G .    
999	. . . . T .    
1000	. . . . A .    
1001	. . . . T .    
1002	. . . . A .    
1003	. . . . C .    
1004	. . . . A .    
1005	. . . . G .    
1006	. . . . T .    
1007	. . . . C .    
1008	. . . . A .    
1009	. . . . T .    
1010	. . . . C .    
1011	. . . . A .    
1012	. . . . A .    
1013	. . . . A .    
1014	. . . . G .    
1015	. . . . C .    
1016	. . . . T .    
1017	. . . . G .    
1018	. . . . A .    
1019	. . . . T .    
1020	. . . . A .    
1021	. . . . T .    
1022	. . . . T .    
1023	. . . . A .    
1024	. . . . A .    
1025	. . . . G .    
1026	. . . . G .    
1027	. . . . C .    
1028	. . . . A .    
1029	. . . . G .    
1030	. . . . T .    
1031	. . . . G .    
1032	. . . . C .    
1033	. . . . A .    
1034	. . . . A .    
1035	. . . . G .    
1036	. . . . C .    
1037	. . . . A .    
1038	. . . . A .    
1039	. . . . T .    
1040	. . . . C .    
1041	. . . . A .    
1042	. . . . T .    
1043	. . . . A .    
1044	. . . . A .    
1045	. . . . C .    
1046	. . . . G .    
1047	. . . . G .    
1048	. . . . A .    
1049	. . . . A .    
1050	. . . . A .    
1051	. . . . G .    
1052	. . . . A .    
1053	. . . . G .    
1054	. . . . C .    
1055	. . . . C .    
1056	. . . . A .    
1057	. . . . T .    
1058	. . . . G .    
1059	. . . . A .    
1060	. . . . A .    
1061	. . . . A .    
1062	. . . . T .    
1063	. . . . G .    
1064	. . . . A .    
1065	. . . . T .    
1066	. . . . T .    
1067	. . . . A .    
1068	. . . . C .    
1069	. . . . G .    
1070	. . . . C .    
1071	. . . . A .    
1072	. . . . A .    
1073	. . . . C .    
1074	. . . . A .    
1075	. . . . C .    
1076	. . . . A .    
1077	. . . . C .    
1078	. . . . A .    
1079	. . . . A .    
1080	. . . . T .    
1081	. . . . A .    
1082	. . . . T .    
1083	. . . . T .    
1084	. . . . G .    
1085	. . . . C .    
1086	. . . . C .    
1087	. . . . T .    
1088	. . . . A .    
1089	. . . . T .    
1090	. . . . G .    
1091	. . . . C .    
1092	. . . . C .    
1093	. . . . G .    
1094	. . . . A .    
1095	. . . . T .    
1096	. . . . T .    
1097	. . . . T .    
1098	. . . . G .    
1099	. . . . A .    
1100	. . . . G .    
1101	. . . . G .    
1102	. . . . T .    
1103	. . . . T .    
1104	. . . . G .    
1105	. . . . A .    
1106	. . . . T .    
1107	. . . . T .    
1108	. . . . T .    
1109	. . . . C .    
1110	. . . . A .    
1111	. . . . A .    
1112	. . . . G .    
1113	. . . . T .    
1114	. . . . G .    
1115	. . . . A .    
1116	. . . . T .    
1117	. . . . T .    
1118	. . . . T .    
1119	. . . . T .    
1120	. . . . T .    
1121	. . . . T .    
1122	. . . . T .    
1123	. . . . T .    
1124	. . . . T .    
1125	. . . . T .    
1126	. . . . T .    
1127	. . . . C .    
1128	. . . . G .    
1129	. . . . T .    
1130	. . . . C .    
1131	. . . . C .    
1132	. . . . A .    
1133	. . . . A .    
1134	. . . . A .    
1135	. . . . A .    
1136	. . . . A .    
1137	. . . . A .    
1138	. . . . C .    
1139	. . . . A .    
1140	. . . . A .    
1141	. . . . A .    
1142	. . . . G .    
1143	. . . . T .    
1144	. . . . T .    
1145	. . . . T .    
1146	. . . . A .    
1147	. . . . T .    
1148	. . . . C .    
1149	. . . . T .    
1150	. . . . A .    
1151	. . . . C .    
1152	. . . . C .    
1153	. . . . G .    
1154	. . . . T .    
1155	. . . . A .    
1156	. . . . C .    
1157	. . . . T .    
1158	. . . . A .    
1159	. . . . A .    
1160	. . . . A .    
1161	. . . . G .    
1162	. . . . T .    
1163	. . . . G .    
1164	. . . . T .    
1165	. . . . C .    
1166	. . . . T .    
1167	. . . . G .    
1168	. . . . T .    
1169	. . . . T .    
1170	. . . . A .    
1171	. . . . A .    
1172	. . . . T .    
1173	. . . . T .    
1174	. . . . G .    
1175	. . . . C .    
1176	. . . . T .    
1177	. . . . A .    
1178	. . . . C .    
1179	. . . . C .    
1180	. . . . T .    
1181	. . . . T .    
1182	. . . . A .    
1183	. . . . A .    
1184	. . . . T .    
1185	. . . . A .    
1186	. . . . C .    
1187	. . . . C .    
1188	. . . . T .    
1189	. . . . G .    
1190	. . . . A .    
1191	. . . . A .    
1192	. . . . A .    
1193	. . . . A .    
1194	. . . . T .    
1195	. . . . A .    
1196	. . . . A .    
1197	. . . . T .    
1198	. . . . A .    
1199	. . . . C .    
1200	. . . . C .    
1201	. . . . A .    
1202	. . . . C .    
1203	. . . . A .    
1204	. . . . G .    
1205	. . . . C .    
1206	. . . . T .    
1207	. . . . G .    
1208	. . . . T .    
1209	. . . . G .    
1210	. . . . G .    
1211	. . . . G .    
1212	. . . . A .    
1213	. . . . A .    
1214	. . . . T .    
1215	. . . . T .    
1216	. . . . G .    
1217	. . . . T .    
1218	. . . . C .    
1219	. . . . C .    
1220	. . . . A .    
1221	. . . . A .    
1222	. . . . T .    
1223	. . . . C .    
1224	. . . . A .    
1225	. . . . G .    
1226	. . . . A .    
1227	. . . . A .    
1228	. . . . T .    
1229	. . . . C .    
1230	. . . . A .    
1231	. . . . G .    
1232	. . . . G .    
1233	. . . . G .    
1234	. . . . C .    
1235	. . . . A .    
1236	. . . . G .    
1237	. . . . C .    
1238	. . . . T .    
1239	. . . . C .    
1240	. . . . C .    
1241	. . . . T .    
1242	. . . . G .    
1243	. . . . A .    
1244	. . . . A .    
1245	. . . . G .    
1246	. . . . A .    
1247	. . . . C .    
1248	. . . . C .    
1249	. . . . G .    
1250	. . . . A .    
1251	. . . . C .    
1252	. . . . T .    
1253	. . . . T .    
1254	. . . . C .    
1255	. . . . A .    
1256	. . . . G .    
1257	. . . . T .    
1258	. . . . G .    
1259	. . . . A .    
1260	. . . . T .    
1261	. . . . T .    
1262	. . . . G .    
1263	. . . . G .    
1264	. . . . T .    
1265	. . . . T .    
1266	. . . . G .    
1267	. . . . T .    
1268	. . . . C .    
1269	. . . . T .    
1270	. . . . C .    
1271	. . . . A .    
1272	. . . . G .    
1273	. . . . A .    
1274	. . . . G .    
1275	. . . . C .    
1276	. . . . A .    
1277	. . . . G .    
1278	. . . . C .    
1279	. . . . T .    
1280	. . . . C .    
1281	. . . . C .    
1282	. . . . T .    
1283	. . . . C .    
1284	. . . . A .    
1285	. . . . A .    
1286	. . . . G .    
1287	. . . . A .    
1288	. . . . C .    
1289	. . . . A .    
1290	. . . . G .    
1291	. . . . A .    
1292	. . . . C .    
1293	. . . . T .    
1294	. . . . T .    
1295	. . . . C .    
1296	. . . . A .    
1297	. . . . G .    
1298	. . . . C .    
1299	. . . . G .    
1300	. . . . A .    
1301	. . . . T .    
1302	. . . . T .    
1303	. . . . G .    
1304	. . . . G .    
1305	. . . . T .    
1306	. . . . T .    
1307	. . . . G .    
1308	. . . . T .    
1309	. . . . C .    
1310	. . . . T .    
1311	. . . . C .    
1312	. . . . A .    
1313	. . . . G .    
1314	. . . . A .    
1315	. . . . G .    
1316	. . . . C .    
1317	. . . . A .    
1318	. . . . G .    
1319	. . . . C .    
1320	. . . . T .    
1321	. . . . C .    
1322	. . . . C .    
1323	. . . . T .    
1324	. . . . C .    
1325	. . . . A .    
1326	. . . . A .    
1327	. . . . G .    
1328	. . . . A .    
1329	. . . . C .    
1330	. . . . A .    
1331	. . . . G .    
1332	. . . . A .    
1333	. . . . C .    
1334	. . . . T .    
1335	. . . . T .    
1336	. . . . C .    
1337	. . . . A .    
1338	. . . . T .    
1339	. . . . C .    
1340	. . . . A .    
1341	. . . . A .    
1342	. . . . T .    
1343	. . . . T .    
1344	. . . . G .    
1345	. . . . G .    
1346	. . . . C .    
1347	. . . . T .    
1348	. . . . G .    
1349	. . . . T .    
1350	. . . . C .    
1351	. . . . T .    
1352	. . . . C .    
1353	. . . . C .    
1354	. . . . A .    
1355	. . . . G .    
1356	. . . . A .    
1357	. . . . A .    
1358	. . . . G .    
1359	. . . . C .    
1360	. . . . A .    
1361	. . . . G .    
1362	. . . . A .    
1363	. . . . A .    
1364	. . . . G .    
1365	. . . . G .    
1366	. . . . A .    
1367	. . . . A .    
1368	. . . . C .    
1369	. . . . T .    
1370	. . . . G .    
1371	. . . . A .    
1372	. . . . A .    
1373	. . . . T .    
1374	. . . . T .    
1375	. . . . C .    
1376	. . . . T .    
1377	. . . . G .    
1378	. . . . G .    
1379	. . . . G .    
1380	. . . . A .    
1381	. . . . A .    
1382	. . . . A .    
1383	. . . . C .    
1384	. . . . T .    
1385	. . . . G .    
1386	. . . . A .    
1387	. . . . A .    
1388	. . . . G .    
1389	. . . . G .    
1390	. . . . T .    
1391	. . . . G .    
1392	. . . . G .    
1393	. . . . C .    
1394	. . . . G .    
1395	. . . . C .    
1396	. . . . T .    
1397	. . . . C .    
1398	. . . . T .    
1399	. . . . G .    
1400	. . . . C .    
1401	. . . . T .    
1402	. . . . C .    
1403	. . . . C .    
1404	. . . . T T    
1405	. . . . T T    
1406	. . . . A A    
1407	. . . . C C    
1408	. . . . T T    
1409	. . . . G G    
1410	. . . . C C    
1411	. . . . G G    
1412	. . . . T T    
1413	. . . . A A    
1414	. . . . A A    
1415	. . . . A A    
1416	. . . . G G    
1417	. . . . G G    
1418	. . . . A A    
1419	. . . . A A    
1420	. . . . A A    
1421	. . . . A A    
1422	. . . . A A    
1423	. . . . C C    
1424	. . . . G G    
1425	. . . . T T    
1426	. . . . G G    
1427	. . . . C C    
1428	. . . . T T    
1429	. . . . C C    
1430	. . . . T T    
1431	. . . . A A    
1432	. . . . C C    
1433	. . . . C C    
1434	. . . . A A    
1435	. . . . G G    
1436	. . . . T T    
1437	. . . . T T    
1438	. . . . C C    
1439	. . . . A A    
1440	. . . . C C    
1441	. . . . T T    
1442	. . . . C C    
1443	. . . . A A    
1444	. . . . G G    
1445	. . . . T T    
1446	. . . . C C    
1447	. . . . G G    
1448	. . . . G G    
1449	. . . . C C    
1450	. . . . C C    
1451	. . . . G G    
1452	. . . . T T    
1453	. . . . A A    
1454	. . . . C C    
1455	. . . . G G    
1456	. . . . A A    
1457	. . . . C C    
1458	. . . . G G    
1459	. . . . T T    
1460	. . . . T T    
1461	. . . . T T    
1462	. . . . T T    
1463	. . . . A A    
1464	. . . . T T    
1465	. . . . T T    
1466	. . . . G G    
1467	. . . . C C    
1468	. . . . C C    
1469	. . . . T T    
1470	. . . . T T    
1471	. . . . T T    
1472	. . . . T T    
1473	. . . . G G    
1474	. . . . A A    
1475	. . . . T T    
1476	. . . . T T    
1477	. . . . G G    
1478	. . . . C C    
1479	. . . . T T    
1480	. . . . C C    
1481	. . . . T T    
1482	. . . . A A    
1483	. . . . G G    
1484	. . . . G G    
1485	. . . . A A    
1486	. . . . T T    
1487	. . . . G G    
1488	. . . . A A    
1489	. . . . G G    
1490	. . . . C C    
1491	. . . . T T    
1492	. . . . C C    
1493	. . . . T T    
1494	. . . . C C    
1495	. . . . C C    
1496	. . . . C T    
1497	. . . . . T    
1498	. . . . . T    
1499	. . . . . G    
1500	. . . . . G    
1501	. . . . . G    
1502	. . . . . A    
1503	. . . . . T    
1504	. . . . . T    
1505	. . . . . G    
1506	. . . . . A    
1507	. . . . . C    
1508	. . . . . T    
1509	. . . . . G    
1510	. . . . . T    
1511	. . . . . G    
1512	. . . . . A    
1513	. . . . . A    
1514	. . . . . T    
1515	. . . . . A    
1516	. . . . . T    
1517	. . . . . C    
1518	. . . . . C    
1519	. . . . . G    
1520	. . . . . A    
1521	. . . . . C    
1522	. . . . . A    
1523	. . . . . C    
1524	. . . . . A    
1525	. . . . . C    
1526	. . . . . A    
1527	. . . . . G    
1528	. . . . . G    
1529	. . . . . G    
1530	. . . . . A    
1531	. . . . . C    
1532	. . . . . A    
1533	. . . . . C    
1534	. . . . . C    
1535	. . . . . C    
1536	. . . . . A    
1537	. . . . . C    
1538	. . . . . T    
1539	. . . . . T    
1540	. . . . . G    
1541	. . . . . C    
1542	. . . . . T    
1543	. . . . . T    
1544	. . . . . G    
1545	. . . . . T    
1546	. . . . . A    
1547	. . . . . G    
1548	. . . . . G    
1549	. . . . . C    
1550	. . . . . A    
1551	. . . . . A    
1552	. . . . . A    
1553	. . . . . C    
1554	. . . . . A    
1555	. . . . . C    
1556	. . . . . A    
1557	. . . . . T    
1558	. . . . . C    
1559	. . . . . A    
1560	. . . . . T    
1561	. . . . . T    
1562	. . . . . T    
1563	. . . . . G    
1564	. . . . . T    
1565	. . . . . T    
1566	. . . . . C    
1567	. . . . . G    
1568	. . . . . G    
1569	. . . . . T    
1570	. . . . . T    
1571	. . . . . T    
1572	. . . . . C    
1573	. . . . . C    
1574	. . . . . A    
1575	. . . . . A    
1576	. . . . . A    
1577	. . . . . G    
1578	. . . . . G    
1579	. . . . . C    
1580	. . . . . T    
1581	. . . . . G    
1582	. . . . . C    
1583	. . . . . A    
1584	. . . . . T    
1585	. . . . . A    
1586	. . . . . G    
1587	. . . . . A    
1588	. . . . . A    
1589	. . . . . G    
1590	. . . . . G    
1591	. . . . . G    
1592	. . . . . A    
1593	. . . . . T    
1594	. . . . . G    
1595	. . . . . T    
1596	. . . . . G    
1597	. . . . . T    
1598	. . . . . G    
1599	. . . . . T    
1600	. . . . . T    
1601	. . . . . T    
1602	. . . . . G    
1603	. . . . . G    
1604	. . . . . G    
1605	. . . . . T    
1606	. . . . . A    
1607	. . . . . G    
1608	. . . . . A    
1609	. . . . . A    
1610	. . . . . G    
1611	. . . . . A    
1612	. . . . . G    
1613	. . . . . G    
1614	. . . . . T    
1615	. . . . . T    
1616	. . . . . C    
1617	. . . . . T    
1618	. . . . . T    
1619	. . . . . G    
1620	. . . . . A    
1621	. . . . . T    
1622	. . . . . G    
1623	. . . . . T    
1624	. . . . . C    
1625	. . . . . T    
1626	. . . . . G    
1627	. . . . . T    
1628	. . . . . G    
1629	. . . . . A    
1630	. . . . . G    
1631	. . . . . G    
1632	. . . . . C    
1633	. . . . . A    
1634	. . . . . T    
1635	. . . . . T    
1636	. . . . . C    
1637	. . . . . A    
1638	. . . . . A    
1639	. . . . . T    
1640	. . . . . C    
1641	. . . . . T    
1642	. . . . . T    
1643	. . . . . G    
1644	. . . . . A    
1645	. . . . . A    
1646	. . . . . C    
1647	. . . . . T    
1648	. . . . . T    
1649	. . . . . C    
1650	. . . . . T    
1651	. . . . . C    
1652	. . . . . T    
1653	. . . . . G    
1654	. . . . . G    
1655	. . . . . C    
1656	. . . . . T    
1657	. . . . . T    
1658	. . . . . C    
1659	. . . . . T    
1660	. . . . . T    
1661	. . . . . T    
1662	. . . . . T    
1663	. . . . . C    
1664	. . . . . A    
1665	. . . . . A    
1666	. . . . . T    
1667	. . . . . T    
1668	. . . . . A    
1669	. . . . . C    
1670	. . . . . C    
1671	. . . . . T    
1672	. . . . . C    
1673	. . . . . C    
1674	. . . . . C    
1675	. . . . . T    
1676	. . . . . A    
1677	. . . . . T    
1678	. . . . . G    
1679	. . . . . A    
1680	. . . . . A    
1681	. . . . . A    
1682	. . . . . G    
1683	. . . . . G    
1684	. . . . . C    
1685	. . . . . A    
1686	. . . . . G    
1687	. . . . . A    
1688	. . . . . G    
1689	. . . . . A    
1690	. . . . . A    
1691	. . . . . G    
1692	. . . . . A    
1693	. . . . . G    
1694	. . . . . G    
1695	. . . . . C    
1696	. . . . . T    
1697	. . . . . G    
1698	. . . . . C    
1699	. . . . . T    
1700	. . . . . A    
1701	. . . . . G    
1702	. . . . . G    
1703	. . . . . A    
1704	. . . . . G    
1705	. . . . . A    
1706	. . . . . G    
1707	. . . . . A    
1708	. . . . . G    
1709	. . . . . C    
1710	. . . . . A    
1711	. . . . . T    
1712	. . . . . G    
1713	. . . . . A    
1714	. . . . . G    
1715	. . . . . G    
1716	. . . . . G    
1717	. . . . . G    
1718	. . . . . T    
1719	. . . . . C    
1720	. . . . . C    
1721	. . . . . T    
1722	. . . . . T    
1723	. . . . . G    
1724	. . . . . A    
1725	. . . . . G    
1726	. . . . . G    
1727	. . . . . C    
1728	. . . . . A    
1729	. . . . . G    
1730	. . . . . G    
1731	. . . . . A    
1732	. . . . . T    
1733	. . . . . G    
1734	. . . . . G    
1735	. . . . . T    
1736	. . . . . T    
1737	. . . . . C    
1738	. . . . . C    
1739	. . . . . C    
1740	. . . . . C    
1741	. . . . . G    
1742	. . . . . G    
1743	. . . . . T    
1744	. . . . . C    
1745	. . . . . G    
1746	. . . . . T    
1747	. . . . . T    
1748	. . . . . C    
1749	. . . . . A    
1750	. . . . . C    
1751	. . . . . C    
1752	. . . . . C    
1753	. . . . . A    
1754	. . . . . G    
1755	. . . . . T    
1756	. . . . . G    
1757	. . . . . C    
1758	. . . . . A    
1759	. . . . . G    
1760	. . . . . G    
1761	. . . . . T    
1762	. . . . . A    
1763	. . . . . C    
1764	. . . . . C    
1765	. . . . . C    
1766	. . . . . T    
1767	. . . . . C    
1768	. . . . . T    
1769	. . . . . A    
1770	. . . . . G    
1771	. . . . . T    
1772	. . . . . C    
1773	. . . . . A    
1774	. . . . . T    
1775	. . . . . G    
1776	. . . . . T    
1777	. . . . . C    
1778	. . . . . G    
1779	. . . . . G    
1780	. . . . . C    
1781	. . . . . C    
1782	. . . . . A    
1783	. . . . . T    
1784	. . . . . G    
1785	. . . . . C    
1786	. . . . . C    
1787	. . . . . G    
1788	. . . . . A    
1789	. . . . . T    
1790	. . . . . G    
1791	. . . . . G    
1792	. . . . . C    
1793	. . . . . C    
1794	. . . . . C    
1795	. . . . . G    
1796	. . . . . C    
1797	. . . . . G    
1798	. . . . . C    
1799	. . . . . C    
1800	. . . . . G    
1801	. . . . . A    
1802	. . . . . A    
1803	. . . . . C    
1804	. . . . . A    
1805	. . . . . G    
1806	. . . . . T    
1807	. . . . . A    
1808	. . . . . C    
1809	. . . . . A    
1810	. . . . . G    
1811	. . . . . G    
1812	. . . . . A    
1813	. . . . . A    
1814	. . . . . C    
1815	. . . . . T    
1816	. . . . . T    
1817	. . . . . G    
1818	. . . . . T    
1819	. . . . . A    
1820	. . . . . G    
1821	. . . . . C    
1822	. . . . . C    
1823	. . . . . A    
1824	. . . . . T    
1825	. . . . . T    
1826	. . . . . C    
1827	. . . . . T    
1828	. . . . . C    
1829	. . . . . A    
1830	. . . . . T    
1831	. . . . . G    
1832	. . . . . C    
1833	. . . . . A    
1834	. . . . . C    
1835	. . . . . C    
1836	. . . . . G    
1837	. . . . . A    
1838	. . . . . G    
1839	. . . . . T    
1840	. . . . . G    
1841	. . . . . G    
1842	. . . . . T    
1843	. . . . . A    
1844	. . . . . G    
1845	. . . . . A    
1846	. . . . . G    
1847	. . . . . C    
1848	. . . . . T    
1849	. . . . . T    
1850	. . . . . A    
1851	. . . . . G    
1852	. . . . . C    
1853	. . . . . G    
1854	. . . . . A    
1855	. . . . . T    
1856	. . . . . G    
1857	. . . . . C    
1858	. . . . . C    
1859	. . . . . T    
1860	. . . . . T    
1861	. . . . . G    
1862	. . . . . A    
1863	. . . . . T    
1864	. . . . . G    
1865	. . . . . A    
1866	. . . . . G    
1867	. . . . . T    
1868	. . . . . C    
1869	. . . . . C    
1870	. . . . . A    
1871	. . . . . G    
1872	. . . . . T    
1873	. . . . . C    
1874	. . . . . T    
1875	. . . . . T    
1876	. . . . . T    
1877	. . . . . C    
1878	. . . . . C    
1879	. . . . . C    
1880	. . . . . A    
1881	. . . . . A    
1882	. . . . . G    
1883	. . . . . C    
1884	. . . . . T    
1885	. . . . . G    
1886	. . . . . A    
1887	. . . . . G    
1888	. . . . . G    
1889	. . . . . G    
1890	. . . . . A    
1891	. . . . . G    
1892	. . . . . G    
1893	. . . . . A    
1894	. . . . . T    
1895	. . . . . T    
1896	. . . . . T    
1897	. . . . . G    
1898	. . . . . G    
1899	. . . . . C    
1900	. . . . . C    
1901	. . . . . A    
1902	. . . . . A    
1903	. . . . . A    
1904	. . . . . C    
1905	. . . . . A    
1906	. . . . . C    
1907	. . . . . A    
1908	. . . . . T    
1909	. . . . . C    
1910	. . . . . T    
1911	. . . . . G    
1912	. . . . . A    
1913	. . . . . C    
1914	. . . . . T    
1915	. . . . . T    
1916	. . . . . G    
1917	. . . . . G    
1918	. . . . . T    
1919	. . . . . G    
1920	. . . . . A    
1921	. . . . . T    
1922	. . . . . G    
1923	. . . . . G    
1924	. . . . . T    
1925	. . . . . G    
1926	. . . . . C    
1927	. . . . . C    
1928	. . . . . G    
1929	. . . . . T    
1930	. . . . . C    
1931	. . . . . G    
1932	. . . . . A    
1933	. . . . . T    
1934	. . . . . G    
1935	. . . . . T    
1936	. . . . . C    
1937	. . . . . A    
1938	. . . . . G    
1939	. . . . . A    
1940	. . . . . G    
1941	. . . . . A    
1942	. . . . . T    
1943	. . . . . A    
1944	. . . . . A    
1945	. . . . . T    
1946	. . . . . G    
1947	. . . . . A    
1948	. . . . . C    
1949	. . . . . T    
1950	. . . . . T    
1951	. . . . . T    
1952	. . . . . G    
1953	. . . . . T    
1954	. . . . . C    
1955	. . . . . A    
1956	. . . . . T    
1957	. . . . . C    
1958	. . . . . C    
1959	. . . . . C    
1960	. . . . . A    
1961	. . . . . G    
1962	. . . . . T    
1963	. . . . . T    
1964	. . . . . C    
1965	. . . . . C    
1966	. . . . . A    
1967	. . . . . C    
1968	. . . . . A    
1969	. . . . . G    
1970	. . . . . G    
1971	. . . . . T    
1972	. . . . . A    
1973	. . . . . G    
1974	. . . . . A    
1975	. . . . . T    
1976	. . . . . G    
1977	. . . . . G    
1978	. . . . . T    
1979	. . . . . G    
1980	. . . . . C    
1981	. . . . . C    
1982	. . . . . C    
1983	. . . . . T    
1984	. . . . . C    
1985	. . . . . G    
1986	. . . . . C    
1987	. . . . . A    
1988	. . . . . G    
1989	. . . . . C    
1990	. . . . . G    
1991	. . . . . A    
1992	. . . . . C    
1993	. . . . . A    
1994	. . . . . C    
1995	. . . . . G    
1996	. . . . . T    
1997	. . . . . G    
1998	. . . . . C    
1999	. . . . . C    
2000	. . . . . C    
2001	. . . . . T    
2002	. . . . . G    
2003	. . . . . G    
2004	. . . . . T    
2005	. . . . . A    
2006	. . . . . C    
2007	. . . . . T    
2008	. . . . . G    
2009	. . . . . A    
2010	. . . . . G    
2011	. . . . . T    
2012	. . . . . G    
2013	. . . . . G    
2014	. . . . . T    
2015	. . . . . G    
2016	. . . . . A    
2017	. . . . . T    
2018	. . . . . G    
2019	. . . . . C    
2020	. . . . . T    
2021	. . . . . G    
2022	. . . . . A    
2023	. . . . . A    
2024	. . . . . A    
2025	. . . . . G    
2026	. . . . . T    
2027	. . . . . A    
2028	. . . . . A    
2029	. . . . . C    
2030	. . . . . G    
2031	. . . . . T    
2032	. . . . . C    
2033	. . . . . A    
2034	. . . . . T    
2035	. . . . . T    
2036	. . . . . A    
2037	. . . . . G    
2038	. . . . . G    
2039	. . . . . C    
2040	. . . . . C    
2041	. . . . . C    
2042	. . . . . C    
2043	. . . . . T    
2044	. . . . . C    
2045	. . . . . C    
2046	. . . . . C    
2047	. . . . . T    
2048	. . . . . C    
2049	. . . . . A    
2050	. . . . . G    
2051	. . . . . C    
2052	. . . . . T    
2053	. . . . . T    
2054	. . . . . C    
2055	. . . . . A    
2056	. . . . . G    
2057	. . . . . A    
2058	. . . . . C    
2059	. . . . . T    
2060	. . . . . C    
2061	. . . . . A    
2062	. . . . . C    
2063	. . . . . T    
2064	. . . . . A    
2065	. . . . . T    
2066	. . . . . C    
2067	. . . . . T    
2068	. . . . . G    
2069	. . . . . G    
2070	. . . . . T    
2071	. . . . . C    
2072	. . . . . A    
2073	. . . . . G    
2074	. . . . . A    
2075	. . . . . T    
2076	. . . . . G    
2077	. . . . . A    
2078	. . . . . G    
2079	. . . . . A    
2080	. . . . . G    
2081	. . . . . G    
2082	. . . . . C    
2083	. . . . . G    
2084	. . . . . G    
2085	. . . . . A    
2086	. . . . . G    
2087	. . . . . A    
2088	. . . . . G    
2089	. . . . . A    
2090	. . . . . C    
2091	. . . . . T    
2092	. . . . . T    
2093	. . . . . C    
2094	. . . . . T    
2095	. . . . . T    
2096	. . . . . G    
2097	. . . . . T    
2098	. . . . . A    
2099	. . . . . G    
2100	. . . . . G    
2101	. . . . . A    
2102	. . . . . G    
2103	. . . . . T    
2104	. . . . . G    
2105	. . . . . T    
2106	. . . . . G    
2107	. . . . . A    
2108	. . . . . T    
2109	. . . . . C    
2110	. . . . . C    
2111	. . . . . G    
2112	. . . . . G    
2113	. . . . . T    
2114	. . . . . T    
2115	. . . . . G    
2116	. . . . . G    
2117	. . . . . T    
2118	. . . . . G    
2119	. . . . . C    
2120	. . . . . T    
2121	. . . . . G    
2122	. . . . . A    
2123	. . . . . A    
2124	. . . . . A    
2125	. . . . . T    
2126	. . . . . C    
2127	. . . . . T    
2128	. . . . . C    
2129	. . . . . T    
2130	. . . . . G    
2131	. . . . . T    
2132	. . . . . C    
2133	. . . . . T    
2134	. . . . . G    
2135	. . . . . C    
2136	. . . . . A    
2137	. . . . . C    
2138	. . . . . A    
2139	. . . . . C    
2140	. . . . . G    
2141	. . . . . C    
2142	. . . . . T    
2143	. . . . . C    
2144	. . . . . A    
2145	. . . . . G    
2146	. . . . . T    
2147	. . . . . A    
2148	. . . . . G    
2149	. . . . . G    
2150	. . . . . T    
2151	. . . . . G    
2152	. . . . . C    
2153	. . . . . C    
2154	. . . . . A    
2155	. . . . . G    
2156	. . . . . T    
2157	. . . . . G    
2158	. . . . . C    
2159	. . . . . A    
2160	. . . . . T    
2161	. . . . . T    
2162	. . . . . C    
2163	. . . . . A    
2164	. . . . . G    
2165	. . . . . C    
2166	. . . . . T    
2167	. . . . . C    
2168	. . . . . T    
2169	. . . . . T    
2170	. . . . . T    
2171	. . . . . A    
2172	. . . . . G    
2173	. . . . . A    
2174	. . . . . T
```
